# Benzyl­tributyl­ammonium 7-hydroxy­naphthalene-1-sulfonate

**DOI:** 10.1107/S1600536809001056

**Published:** 2009-01-17

**Authors:** Yohei Sato, Kazuya Uta, Jin Mizuguchi

**Affiliations:** a Department of Applied Physics, Graduate School of Engineering, Yokohama National University, 79-5 Tokiwadai, Hodogaya-ku, 240-8501 Yokohama, Japan

## Abstract

The title compound, C_19_H_34_N^+^·C_10_H_7_O_4_S^−^, is a charge-control agent used for toners in electrophotography. The anions form one-dimensional chains by O—H⋯O hydrogen bonds in a zigzag fashion along the *c* axis between the OH group of one anion and the sulfonate O atom of a neighboring anion. One of the *n*-butyl chains of the cation is disordered over two sites in a 0.77:0.23 ratio.

## Related literature

For the function of charge-control agents, see: Nash *et al.* (2001 ▶) and for the structure of benzyl­tributyl­ammonium 4-hydroxy­naphthalene-1-sulfonate, benzyl­tributyl­ammonium 6-hydroxy­naphthalene-2-sulfonate, and benzyl­tributyl­ammonium 4-hydroxy­naphthalene-2-sulfonate see: Mizuguchi *et al.* (2007 ▶), Uta *et al.* (2009 ▶), and Uta & Mizuguchi (2009 ▶), respectively.
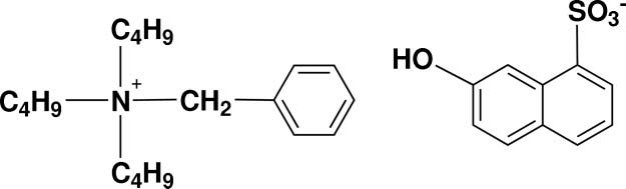


## Experimental

### 

#### Crystal data

C_19_H_34_N^+^·C_10_H_7_O_4_S^−^
*M* *_r_* = 499.70
Monoclinic, 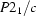

*a* = 19.8286 (6) Å
*b* = 8.8549 (2) Å
*c* = 16.7501 (4) Å
β = 104.7570 (13)°
*V* = 2843.98 (13) Å^3^
*Z* = 4
Cu *K*α radiation
μ = 1.27 mm^−1^
*T* = 296.1 K
0.39 × 0.36 × 0.04 mm

#### Data collection

Rigaku R-AXIS RAPID diffractometer
Absorption correction: multi-scan (*ABSCOR*; Higashi, 1995 ▶) *T*_min_ = 0.650, *T*_max_ = 0.951
24641 measured reflections
5082 independent reflections
2591 reflections with *F* ^2^ > 2σ(*F* ^2^)
*R*_int_ = 0.042

#### Refinement

*R*[*F* ^2^ > 2σ(*F* ^2^)] = 0.080
*wR*(*F* ^2^) = 0.291
*S* = 1.02
5082 reflections
328 parameters
H-atom parameters constrained
Δρ_max_ = 0.22 e Å^−3^
Δρ_min_ = −0.71 e Å^−3^

### 

Data collection: *PROCESS-AUTO* (Rigaku, 1998 ▶); cell refinement: *PROCESS-AUTO*; data reduction: *CrystalStructure* (Rigaku/MSC, 2006 ▶); program(s) used to solve structure: *SIR2004* (Burla *et al.*, 2003 ▶); program(s) used to refine structure: *SHELXL97* (Sheldrick, 2008 ▶); molecular graphics: *ORTEPIII* (Burnett & Johnson, 1996 ▶); software used to prepare material for publication: *CrystalStructure*.

## Supplementary Material

Crystal structure: contains datablocks global, I. DOI: 10.1107/S1600536809001056/bt2845sup1.cif

Structure factors: contains datablocks I. DOI: 10.1107/S1600536809001056/bt2845Isup2.hkl

Additional supplementary materials:  crystallographic information; 3D view; checkCIF report

## Figures and Tables

**Table 1:** Hydrogen-bond geometry (Å, °)

*D*—H⋯*A*	*D*—H	H⋯*A*	*D*⋯*A*	*D*—H⋯*A*
O4—H4*O*⋯O1^i^	0.82	1.91	2.729 (3)	173

Symmetry code: (i) 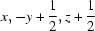.

**Fig. 1.**
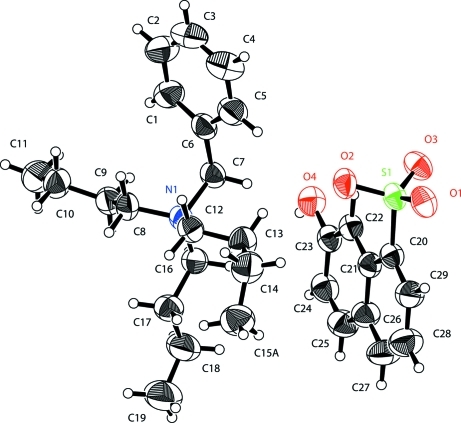
The asymmetric unit of the title compound, showing 30% probability displacement ellipsoids and only the major component of the disordered atoms.

**Fig. 2.**
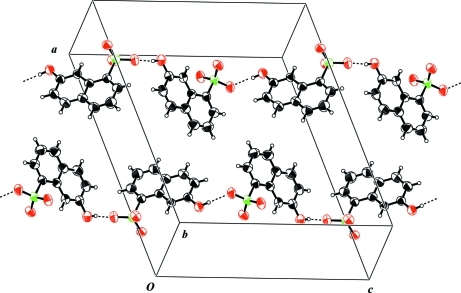
View of the hydrogen-bonded (dashed lines) chain running along the c axis. Only anions are shown for clarity.

## supplementary crystallographic information

### Comment

The title compound is a charge-control-agent used for toners in electrophotography. The background of the present study has been set out in our previous paper (Uta *et al.*, 2009). We have previously investigated the crystal structure of three isomers of compound (I) in connection with the mechanism of their high melting points [benzyltributylammonium 4-hydroxynaphthalene-1-sulfonate (Mizuguchi *et al.*,  2007); benzyltributylammonium 6-hydroxynaphthalene-2-sulfonate (Uta *et al.*, 2009); benzyltributylammonium 4-hydroxynaphthalene-4-sulfonate (Uta & Mizuguchi, 2009)]. The anions in the  two former isomers are found to form chains of O—H···O intermolecular hydrogen bonds between the OH group of one anion and the sulfonate O atom of the neighboring one. The present hydrogen-bond network ensures a high thermal stability of these compounds as characterized by the melting points of 462 and 433K, respectively. On the other hand, the last isomer was characterized by a hydrogen-bonded dimer of the anions through O–H···O hydrogen bonding (melting point: 451 K). The present paper describes again one-dimensional chains of O—H···O intermoleclar hydrogen bonds.

Fig. 1 shows the *ORTEPIII* plot   of the title compound. The ions have no crystallographically imposed symmetry. Fig. 2 shows a hydrogen-bonded chain along the *c* axis between the OH group of one anion and the sulfonic O atom of the neighboring one. The present linear chain is typically characterized by a zigzag form which is simlar to the one in benzyltributylammonium 4-hydroxynaphthalene-1-sulfonate (Mizuguchi *et al.*, 2007) rather than the one in benzyltributylammonium 6-hydroxynaphthalene-2-sulfonate, Uta *et al.*, 2009).

### Experimental

The title compound was obtained from Orient Chemical Industries, Ltd, and was recrystallized from an acetone solution. After 48 h, a number of colorless crystals were obtained in the form of platelets. The title compound has a melting point of 439 K.

### Refinement

C15 was found to be disordered over two sites. The site occupancies for C15A/C15B refined to 0.77 (1)/0.23 (1). These atoms were refined anisotropically. All H atoms were placed in geometrically idealized positions and constrained to ride on their parent atoms, with C—H = 0.93 (aromatic), 0.96 (methyl), or 0.97Å (methylene), and O—H = 0.82Å, and with *U*_iso_(H) = 1.2*U*_eq_(parent atom).

### Crystal data

**Table:**

C_19_H_34_N^+^·C_10_H_7_O_4_S^−^	*F*(000) = 1080.00
*M**_r_* = 499.70	*D*_x_ = 1.167 Mg m^−^^3^
Monoclinic, *P*2_1_/*c*	Melting point: 439 K
Hall symbol: -P 2ybc	Cu *K*α radiation, λ = 1.54187 Å
*a* = 19.8286 (6) Å	Cell parameters from 15029 reflections
*b* = 8.8549 (2) Å	θ = 3.1–68.1°
*c* = 16.7501 (4) Å	µ = 1.26 mm^−^^1^
β = 104.7570 (13)°	*T* = 296 K
*V* = 2843.98 (13) Å^3^	Platelet, colorless
*Z* = 4	0.39 × 0.36 × 0.04 mm

### Data collection

**Table:**

Rigaku R-AXIS RAPID diffractometer	2591 reflections with *F*^2^ > 2σ(*F*^2^)
Detector resolution: 10.00 pixels mm^-1^	*R*_int_ = 0.042
ω scans	θ_max_ = 68.2°
Absorption correction: multi-scan (*ABSCOR*; Higashi, 1995)	*h* = −23→23
*T*_min_ = 0.650, *T*_max_ = 0.951	*k* = −10→10
24641 measured reflections	*l* = −19→19
5082 independent reflections	

### Refinement

**Table:**

Refinement on *F*^2^	H-atom parameters constrained
*R*[*F*^2^ > 2σ(*F*^2^)] = 0.080	*w* = 1/[σ^2^(*F*_o_^2^) + (0.1837*P*)^2^ + 0.0926*P*] where *P* = (*F*_o_^2^ + 2*F*_c_^2^)/3
*wR*(*F*^2^) = 0.291	(Δ/σ)_max_ < 0.001
*S* = 1.02	Δρ_max_ = 0.22 e Å^−^^3^
5082 reflections	Δρ_min_ = −0.71 e Å^−^^3^
328 parameters	

### Special details

**Table:**

Geometry. All e.s.d.'s (except the e.s.d. in the dihedral angle between two l.s. planes) are estimated using the full covariance matrix. The cell e.s.d.'s are taken into account individually in the estimation of e.s.d.'s in distances, angles and torsion angles; correlations between e.s.d.'s in cell parameters are only used when they are defined by crystal symmetry. An approximate (isotropic) treatment of cell e.s.d.'s is used for estimating e.s.d.'s involving l.s. planes.
Refinement. Refinement of *F*^2^ against ALL reflections. The weighted *R*-factor *wR* and goodness of fit *S* are based on *F*^2^, conventional *R*-factors *R* are based on *F*, with *F* set to zero for negative *F*^2^. The threshold expression of *F*^2^ > σ(*F*^2^) is used only for calculating *R*-factors(gt) *etc*. and is not relevant to the choice of reflections for refinement. *R*-factors based on *F*^2^ are statistically about twice as large as those based on *F*, and *R*- factors based on ALL data will be even larger.

### Fractional atomic coordinates and isotropic or equivalent isotropic displacement parameters (Å^2^)

**Table:**

	*x*	*y*	*z*	*U*_iso_*/*U*_eq_	Occ. (<1)
S1	0.24745 (5)	0.42482 (10)	0.48273 (6)	0.0825 (3)	
O1	0.26341 (15)	0.4863 (3)	0.40868 (16)	0.0995 (8)	
O2	0.20862 (14)	0.5287 (2)	0.51959 (17)	0.1016 (8)	
O3	0.21686 (15)	0.2769 (2)	0.46891 (16)	0.0996 (8)	
O4	0.23117 (15)	0.1438 (3)	0.75592 (16)	0.1015 (8)	
N1	0.19605 (14)	0.5894 (2)	0.83330 (18)	0.0780 (8)	
C1	0.0228 (2)	0.5086 (5)	0.7653 (3)	0.1169 (14)	
C2	−0.0426 (2)	0.5488 (7)	0.7173 (5)	0.143 (2)	
C3	−0.0533 (3)	0.5852 (6)	0.6376 (4)	0.140 (2)	
C4	0.0020 (3)	0.5824 (6)	0.6029 (4)	0.148 (2)	
C5	0.0682 (2)	0.5447 (6)	0.6497 (3)	0.1181 (14)	
C6	0.0800 (2)	0.5085 (4)	0.7324 (2)	0.0913 (11)	
C7	0.1511 (2)	0.4632 (3)	0.7827 (2)	0.0884 (10)	
C8	0.15890 (19)	0.6643 (3)	0.8909 (2)	0.0859 (10)	
C9	0.1472 (2)	0.5662 (4)	0.9600 (2)	0.0980 (11)	
C10	0.1022 (2)	0.6408 (5)	1.0078 (3)	0.1144 (14)	
C11	0.0887 (2)	0.5487 (6)	1.0747 (3)	0.1406 (18)	
C12	0.21187 (19)	0.7113 (4)	0.7765 (2)	0.0874 (10)	
C13	0.2501 (2)	0.6592 (5)	0.7148 (3)	0.1122 (13)	
C14	0.2834 (2)	0.7853 (6)	0.6794 (3)	0.1304 (15)	
C15A	0.3514 (3)	0.8302 (9)	0.7319 (6)	0.144	0.77
C15B	0.3524 (8)	0.7787 (3)	0.6626 (18)	0.132	0.23
C16	0.26209 (19)	0.5147 (4)	0.8810 (2)	0.0901 (10)	
C17	0.3155 (2)	0.6162 (4)	0.9349 (2)	0.1037 (12)	
C18	0.3768 (2)	0.5210 (7)	0.9851 (4)	0.160 (2)	
C19	0.4343 (3)	0.6114 (8)	1.0346 (5)	0.201 (3)	
C20	0.32863 (19)	0.4039 (3)	0.5553 (2)	0.0806 (9)	
C21	0.33489 (19)	0.3335 (3)	0.6343 (2)	0.0819 (9)	
C22	0.27822 (19)	0.2672 (3)	0.6588 (2)	0.0805 (9)	
C23	0.2876 (2)	0.2048 (4)	0.7355 (2)	0.0850 (9)	
C24	0.3539 (2)	0.2060 (5)	0.7924 (2)	0.1034 (12)	
C25	0.4082 (2)	0.2663 (5)	0.7697 (3)	0.1114 (13)	
C26	0.4020 (2)	0.3316 (4)	0.6916 (2)	0.0989 (11)	
C27	0.4596 (2)	0.3959 (6)	0.6690 (3)	0.1288 (17)	
C28	0.4519 (2)	0.4577 (6)	0.5935 (4)	0.1329 (17)	
C29	0.3867 (2)	0.4611 (5)	0.5372 (2)	0.1058 (12)	
H1	0.0283	0.4821	0.8203	0.139*	
H2	−0.0796	0.5492	0.7424	0.170*	
H3	−0.0973	0.6106	0.6067	0.167*	
H4	−0.0045	0.6068	0.5484	0.177*	
H4O	0.2434	0.1103	0.8032	0.121*	
H5	0.1055	0.5422	0.6251	0.141*	
H7A	0.1765	0.4202	0.7459	0.105*	
H7B	0.1458	0.3836	0.8209	0.105*	
H8A	0.1143	0.7008	0.8585	0.101*	
H8B	0.1861	0.7519	0.9151	0.101*	
H9A	0.1250	0.4721	0.9369	0.117*	
H9B	0.1916	0.5411	0.9975	0.117*	
H10A	0.0577	0.6663	0.9691	0.136*	
H10B	0.1241	0.7358	1.0294	0.136*	
H11A	0.0687	0.4516	1.0548	0.207*	
H11B	0.0590	0.5981	1.1033	0.207*	
H11C	0.1333	0.5261	1.1159	0.207*	
H12A	0.2382	0.7901	0.8102	0.103*	
H12B	0.1674	0.7549	0.7463	0.103*	
H13A	0.2859	0.5872	0.7415	0.132*	
H13B	0.2183	0.6079	0.6697	0.132*	
H14A	0.2892	0.7545	0.6259	0.157*	0.77
H14B	0.2526	0.8721	0.6708	0.157*	0.77
H14C	0.2510	0.8117	0.6274	0.157*	0.23
H14D	0.2847	0.8710	0.7158	0.157*	0.23
H15A	0.3817	0.7439	0.7426	0.216*	0.77
H15B	0.3456	0.8696	0.7831	0.216*	0.77
H15C	0.3715	0.9065	0.7043	0.216*	0.77
H15D	0.3827	0.7163	0.7032	0.198*	0.23
H15E	0.3715	0.8787	0.6649	0.198*	0.23
H15F	0.3481	0.7368	0.6087	0.198*	0.23
H16A	0.2833	0.4647	0.8418	0.106*	
H16B	0.2495	0.4361	0.9152	0.106*	
H17A	0.2948	0.6715	0.9730	0.124*	
H17B	0.3325	0.6892	0.9015	0.124*	
H18A	0.3603	0.4528	1.0208	0.188*	
H18B	0.3948	0.4591	0.9465	0.188*	
H19A	0.4543	0.6724	0.9988	0.294*	
H19B	0.4697	0.5462	1.0666	0.294*	
H19C	0.4170	0.6762	1.0708	0.294*	
H22	0.2340	0.2664	0.6214	0.095*	
H24	0.3590	0.1649	0.8447	0.124*	
H25	0.4518	0.2655	0.8078	0.134*	
H27	0.5033	0.3957	0.7065	0.152*	
H28	0.4906	0.4970	0.5786	0.157*	
H29	0.3824	0.5040	0.4856	0.126*	

### Atomic displacement parameters (Å^2^)

**Table:**

	*U*^11^	*U*^22^	*U*^33^	*U*^12^	*U*^13^	*U*^23^
S1	0.1011 (7)	0.0675 (5)	0.0765 (6)	0.0067 (4)	0.0179 (5)	−0.0039 (4)
O1	0.135 (2)	0.0933 (17)	0.0707 (16)	−0.0029 (15)	0.0269 (14)	0.0052 (13)
O2	0.1169 (19)	0.0935 (16)	0.0903 (18)	0.0318 (15)	0.0192 (15)	−0.0127 (14)
O3	0.1196 (19)	0.0722 (14)	0.0969 (19)	−0.0055 (13)	0.0089 (15)	−0.0042 (13)
O4	0.1150 (19)	0.1011 (18)	0.0881 (19)	0.0086 (15)	0.0251 (15)	0.0080 (14)
N1	0.0851 (18)	0.0645 (15)	0.083 (2)	−0.0028 (13)	0.0197 (15)	−0.0066 (13)
C1	0.099 (3)	0.132 (3)	0.122 (3)	−0.014 (2)	0.034 (2)	−0.002 (3)
C2	0.093 (3)	0.157 (4)	0.174 (6)	−0.011 (3)	0.025 (3)	−0.018 (4)
C3	0.108 (3)	0.146 (4)	0.141 (5)	−0.017 (3)	−0.015 (3)	0.000 (4)
C4	0.129 (4)	0.162 (5)	0.135 (5)	−0.025 (3)	−0.002 (4)	0.007 (3)
C5	0.115 (3)	0.141 (3)	0.097 (3)	−0.021 (2)	0.023 (2)	−0.011 (3)
C6	0.099 (2)	0.080 (2)	0.094 (3)	−0.0181 (19)	0.023 (2)	−0.017 (2)
C7	0.096 (2)	0.0723 (19)	0.095 (2)	−0.0084 (18)	0.022 (2)	−0.0098 (19)
C8	0.102 (2)	0.0730 (19)	0.090 (2)	−0.0015 (18)	0.037 (2)	−0.0101 (18)
C9	0.105 (2)	0.098 (2)	0.094 (3)	−0.009 (2)	0.032 (2)	−0.008 (2)
C10	0.119 (3)	0.120 (3)	0.120 (3)	−0.018 (2)	0.060 (2)	−0.011 (2)
C11	0.136 (3)	0.171 (4)	0.129 (4)	−0.033 (3)	0.059 (3)	−0.001 (3)
C12	0.099 (2)	0.0720 (19)	0.092 (2)	−0.0100 (18)	0.025 (2)	−0.0001 (18)
C13	0.128 (3)	0.107 (2)	0.114 (3)	−0.013 (2)	0.054 (2)	0.000 (2)
C14	0.134 (3)	0.119 (3)	0.150 (4)	−0.021 (2)	0.057 (2)	0.002 (2)
C15A	0.128 (4)	0.121 (4)	0.180 (8)	−0.012 (3)	0.034 (4)	−0.007 (5)
C15B	0.144 (10)	0.138 (13)	0.132 (14)	−0.039 (9)	0.069 (10)	−0.023 (12)
C16	0.087 (2)	0.081 (2)	0.099 (2)	0.0024 (19)	0.018 (2)	−0.001 (2)
C17	0.095 (2)	0.101 (2)	0.104 (3)	0.008 (2)	0.005 (2)	0.004 (2)
C18	0.115 (3)	0.158 (4)	0.178 (5)	0.012 (3)	−0.017 (3)	−0.048 (4)
C19	0.130 (4)	0.204 (6)	0.228 (8)	0.025 (4)	−0.028 (4)	−0.061 (6)
C20	0.089 (2)	0.0734 (19)	0.079 (2)	0.0018 (16)	0.0193 (18)	−0.0023 (17)
C21	0.087 (2)	0.0720 (19)	0.085 (2)	0.0074 (17)	0.0179 (19)	−0.0082 (17)
C22	0.091 (2)	0.0735 (19)	0.074 (2)	0.0125 (17)	0.0158 (18)	0.0022 (17)
C23	0.097 (2)	0.075 (2)	0.081 (2)	0.0097 (18)	0.018 (2)	−0.0014 (18)
C24	0.118 (3)	0.101 (2)	0.083 (2)	0.014 (2)	0.011 (2)	0.003 (2)
C25	0.105 (3)	0.120 (3)	0.097 (3)	0.006 (2)	0.002 (2)	0.007 (2)
C26	0.089 (2)	0.105 (2)	0.095 (2)	0.008 (2)	0.009 (2)	0.005 (2)
C27	0.084 (2)	0.175 (5)	0.117 (4)	−0.003 (2)	0.006 (2)	0.017 (3)
C28	0.106 (3)	0.163 (4)	0.130 (4)	−0.019 (3)	0.030 (3)	0.009 (3)
C29	0.097 (2)	0.115 (3)	0.103 (3)	−0.004 (2)	0.020 (2)	−0.002 (2)

### Geometric parameters (Å, °)

**Table:**

S1—O1	1.461 (3)	C5—H5	0.934
S1—O2	1.436 (3)	C7—H7A	0.968
S1—O3	1.438 (2)	C7—H7B	0.976
S1—C20	1.762 (3)	C8—H8A	0.968
O4—C23	1.362 (5)	C8—H8B	0.973
N1—C7	1.542 (4)	C9—H9A	0.974
N1—C8	1.508 (5)	C9—H9B	0.968
N1—C12	1.523 (4)	C10—H10A	0.980
N1—C16	1.503 (4)	C10—H10B	0.973
C1—C2	1.386 (7)	C11—H11A	0.970
C1—C6	1.383 (7)	C11—H11B	0.953
C2—C3	1.337 (11)	C11—H11C	0.993
C3—C4	1.366 (11)	C12—H12A	0.963
C4—C5	1.387 (7)	C12—H12B	0.978
C5—C6	1.383 (7)	C13—H13A	0.974
C6—C7	1.500 (5)	C13—H13B	0.966
C8—C9	1.513 (5)	C14—H14A	0.970
C9—C10	1.495 (6)	C14—H14B	0.970
C10—C11	1.466 (7)	C14—H14C	0.970
C12—C13	1.501 (6)	C14—H14D	0.970
C13—C14	1.496 (7)	C15A—H15A	0.960
C14—C15A	1.465	C15A—H15B	0.960
C14—C15B	1.465	C15A—H15C	0.960
C16—C17	1.502 (5)	C15B—H15D	0.960
C17—C18	1.542 (6)	C15B—H15E	0.960
C18—C19	1.465 (8)	C15B—H15F	0.960
C20—C21	1.440 (5)	C16—H16A	0.972
C20—C29	1.361 (6)	C16—H16B	0.973
C21—C22	1.418 (5)	C17—H17A	0.974
C21—C26	1.429 (5)	C17—H17B	0.969
C22—C23	1.367 (5)	C18—H18A	0.965
C23—C24	1.414 (5)	C18—H18B	0.981
C24—C25	1.341 (7)	C19—H19A	0.965
C25—C26	1.407 (6)	C19—H19B	0.960
C26—C27	1.412 (7)	C19—H19C	0.960
C27—C28	1.351 (8)	C22—H22	0.938
C28—C29	1.393 (6)	C24—H24	0.930
O4—H4O	0.821	C25—H25	0.934
C1—H1	0.930	C27—H27	0.931
C2—H2	0.934	C28—H28	0.931
C3—H3	0.922	C29—H29	0.927
C4—H4	0.915		
			
O1···O4^i^	2.729 (3)	O3···H9B^i^	2.920
O4···O1^ii^	2.729 (3)	O3···H16B^i^	2.254
S1···H3^iii^	2.989	O4···H7A	2.665
O1···H4O^i^	1.912	O4···H14B^v^	2.883
O1···H8B^iv^	2.796	O4···H14D^v^	2.789
O1···H12A^iv^	2.544	C15A···H27^vi^	2.861
O1···H24^i^	2.751	C15B···H19C^iv^	2.275
O2···H3^iii^	2.914	C19···H15E^vii^	2.778
O2···H8B^iv^	2.576	C19···H15F^vii^	2.712
O2···H10B^iv^	2.706	C23···H7A	2.952
O2···H13B	2.571	C23···H14D^v^	2.973
O2···H14A	2.876	C23···H16A	2.924
O3···H3^iii^	2.586	C24···H15B^v^	2.986
O3···H7B^i^	2.893	C24···H16A	2.913
O3···H9A^i^	2.822	C29···H15F	2.906
			
O1—S1—O2	112.46 (16)	C9—C10—H10B	108.1
O1—S1—O3	112.13 (16)	C11—C10—H10A	108.9
O1—S1—C20	105.50 (18)	C11—C10—H10B	110.4
O2—S1—O3	113.73 (18)	H10A—C10—H10B	106.8
O2—S1—C20	105.46 (17)	C10—C11—H11A	111.7
O3—S1—C20	106.79 (15)	C10—C11—H11B	112.6
C7—N1—C8	111.2 (2)	C10—C11—H11C	109.8
C7—N1—C12	110.7 (2)	H11A—C11—H11B	109.2
C7—N1—C16	106.1 (2)	H11A—C11—H11C	106.0
C8—N1—C12	107.4 (2)	H11B—C11—H11C	107.2
C8—N1—C16	110.8 (2)	N1—C12—H12A	108.4
C12—N1—C16	110.7 (2)	N1—C12—H12B	107.6
C2—C1—C6	120.7 (5)	C13—C12—H12A	109.4
C1—C2—C3	121.8 (6)	C13—C12—H12B	108.1
C2—C3—C4	118.7 (5)	H12A—C12—H12B	107.4
C3—C4—C5	120.7 (6)	C12—C13—H13A	108.8
C4—C5—C6	121.1 (5)	C12—C13—H13B	109.8
C1—C6—C5	116.9 (3)	C14—C13—H13A	109.1
C1—C6—C7	121.7 (4)	C14—C13—H13B	107.9
C5—C6—C7	121.3 (4)	H13A—C13—H13B	107.8
N1—C7—C6	116.4 (2)	C13—C14—H14A	108.9
N1—C8—C9	115.6 (2)	C13—C14—H14B	108.9
C8—C9—C10	112.6 (3)	C13—C14—H14C	106.1
C9—C10—C11	114.4 (4)	C13—C14—H14D	106.1
N1—C12—C13	115.6 (3)	C15A—C14—H14A	108.9
C12—C13—C14	113.3 (3)	C15A—C14—H14B	108.9
C13—C14—C15A	113.3	C15B—C14—H14C	106.1
C13—C14—C15B	125.1	C15B—C14—H14D	106.1
N1—C16—C17	116.2 (2)	H14A—C14—H14B	107.7
C16—C17—C18	109.7 (3)	H14C—C14—H14D	106.3
C17—C18—C19	113.7 (5)	C14—C15A—H15A	109.5
S1—C20—C21	122.0 (2)	C14—C15A—H15B	109.5
S1—C20—C29	118.9 (3)	C14—C15A—H15C	109.5
C21—C20—C29	119.1 (3)	H15A—C15A—H15B	109.5
C20—C21—C22	123.8 (3)	H15A—C15A—H15C	109.5
C20—C21—C26	118.0 (3)	H15B—C15A—H15C	109.5
C22—C21—C26	118.2 (3)	C14—C15B—H15D	109.5
C21—C22—C23	120.8 (3)	C14—C15B—H15E	109.5
O4—C23—C22	118.2 (3)	C14—C15B—H15F	109.5
O4—C23—C24	121.1 (3)	H15D—C15B—H15E	109.5
C22—C23—C24	120.7 (4)	H15D—C15B—H15F	109.5
C23—C24—C25	119.2 (4)	H15E—C15B—H15F	109.5
C24—C25—C26	122.8 (3)	N1—C16—H16A	107.9
C21—C26—C25	118.3 (4)	N1—C16—H16B	108.0
C21—C26—C27	119.6 (4)	C17—C16—H16A	108.6
C25—C26—C27	122.0 (3)	C17—C16—H16B	108.8
C26—C27—C28	120.5 (4)	H16A—C16—H16B	107.0
C27—C28—C29	120.5 (5)	C16—C17—H17A	110.1
C20—C29—C28	122.2 (4)	C16—C17—H17B	110.4
C23—O4—H4O	109.0	C18—C17—H17A	108.8
C2—C1—H1	119.7	C18—C17—H17B	109.7
C6—C1—H1	119.6	H17A—C17—H17B	108.0
C1—C2—H2	117.8	C17—C18—H18A	109.5
C3—C2—H2	120.3	C17—C18—H18B	108.4
C2—C3—H3	120.5	C19—C18—H18A	109.3
C4—C3—H3	120.8	C19—C18—H18B	108.5
C3—C4—H4	119.7	H18A—C18—H18B	107.3
C5—C4—H4	119.6	C18—C19—H19A	109.8
C4—C5—H5	119.9	C18—C19—H19B	109.9
C6—C5—H5	119.1	C18—C19—H19C	109.4
N1—C7—H7A	108.2	H19A—C19—H19B	109.1
N1—C7—H7B	107.9	H19A—C19—H19C	109.1
C6—C7—H7A	108.3	H19B—C19—H19C	109.5
C6—C7—H7B	108.6	C21—C22—H22	118.9
H7A—C7—H7B	107.1	C23—C22—H22	120.3
N1—C8—H8A	108.3	C23—C24—H24	119.4
N1—C8—H8B	108.0	C25—C24—H24	121.4
C9—C8—H8A	108.9	C24—C25—H25	118.2
C9—C8—H8B	108.4	C26—C25—H25	119.0
H8A—C8—H8B	107.4	C26—C27—H27	119.6
C8—C9—H9A	109.2	C28—C27—H27	119.8
C8—C9—H9B	109.7	C27—C28—H28	119.7
C10—C9—H9A	108.7	C29—C28—H28	119.8
C10—C9—H9B	108.8	C20—C29—H29	118.5
H9A—C9—H9B	107.7	C28—C29—H29	119.3
C9—C10—H10A	107.9	C14—H14A—C15B	80.7
			
O1—S1—C20—C21	−174.5 (2)	C8—C9—C10—C11	179.1 (3)
O1—S1—C20—C29	8.0 (3)	N1—C12—C13—C14	−162.1 (3)
O2—S1—C20—C21	66.3 (3)	C12—C13—C14—C15A	82.9
O2—S1—C20—C29	−111.2 (3)	C12—C13—C14—C15B	140.1
O3—S1—C20—C21	−55.1 (3)	N1—C16—C17—C18	175.1 (4)
O3—S1—C20—C29	127.5 (3)	C16—C17—C18—C19	175.3 (5)
C7—N1—C8—C9	66.7 (3)	S1—C20—C21—C22	4.1 (5)
C8—N1—C7—C6	56.5 (4)	S1—C20—C21—C26	−175.4 (2)
C7—N1—C12—C13	−59.3 (3)	S1—C20—C29—C28	175.7 (4)
C12—N1—C7—C6	−62.8 (4)	C21—C20—C29—C28	−1.8 (6)
C7—N1—C16—C17	179.2 (3)	C29—C20—C21—C22	−178.5 (3)
C16—N1—C7—C6	177.1 (3)	C29—C20—C21—C26	2.1 (5)
C8—N1—C12—C13	179.1 (2)	C20—C21—C22—C23	−178.7 (3)
C12—N1—C8—C9	−172.1 (2)	C20—C21—C26—C25	178.5 (3)
C8—N1—C16—C17	−60.0 (4)	C20—C21—C26—C27	−0.9 (6)
C16—N1—C8—C9	−51.0 (3)	C22—C21—C26—C25	−1.0 (5)
C12—N1—C16—C17	59.1 (4)	C22—C21—C26—C27	179.6 (4)
C16—N1—C12—C13	58.0 (3)	C26—C21—C22—C23	0.7 (5)
C2—C1—C6—C5	−2.3 (6)	C21—C22—C23—O4	179.5 (3)
C2—C1—C6—C7	−179.1 (4)	C21—C22—C23—C24	0.5 (5)
C6—C1—C2—C3	1.7 (8)	O4—C23—C24—C25	179.6 (4)
C1—C2—C3—C4	0.0 (8)	C22—C23—C24—C25	−1.5 (6)
C2—C3—C4—C5	−1.0 (9)	C23—C24—C25—C26	1.2 (7)
C3—C4—C5—C6	0.2 (6)	C24—C25—C26—C21	0.0 (5)
C4—C5—C6—C1	1.4 (7)	C24—C25—C26—C27	179.4 (4)
C4—C5—C6—C7	178.2 (4)	C21—C26—C27—C28	−0.6 (7)
C1—C6—C7—N1	−87.5 (4)	C25—C26—C27—C28	−180.0 (4)
C5—C6—C7—N1	95.9 (4)	C26—C27—C28—C29	1.0 (9)
N1—C8—C9—C10	−172.6 (2)	C27—C28—C29—C20	0.2 (7)

Symmetry codes: (i) *x*, −*y*+1/2, *z*−1/2; (ii) *x*, −*y*+1/2, *z*+1/2; (iii) −*x*, −*y*+1, −*z*+1; (iv) *x*, −*y*+3/2, *z*−1/2; (v) *x*, *y*−1, *z*; (vi) −*x*+1, *y*+1/2, −*z*+3/2; (vii) *x*, −*y*+3/2, *z*+1/2.

### Hydrogen-bond geometry (Å, °)

**Table:**

*D*—H···*A*	*D*—H	H···*A*	*D*···*A*	*D*—H···*A*
O4—H4O···O1^ii^	0.82	1.91	2.729 (3)	173

Symmetry codes: (ii) *x*, −*y*+1/2, *z*+1/2.
